# Shall the Society Publish a Journal?

**Published:** 1904-03

**Authors:** 


### Shall the Society Publish a Journal ?

THE Medical Society of the State of New York should ask itself this question in all seriousness. It should be answered upon plain business principles, devoid of prejudice, without bias, and uninfluenced by considerations other than those which should govern similar individual enterprises.

In a review of the last volume of the transactions of the society published in the December (1903) issue, the JOURNAL said:

The advantages of an annual volume of society transactions are so superior to the journal method, it is surprising that it is not adhered to, at least by the older societies. Journals become lost, mislaid, or otherwise disposed of, and thus the record is broken; whereas, a book properly bound is always accessible, and is an ornament to the library. It is the cheaper method, too; hence, it does not necessitate large fees. The journal method will bankrupt any society in the end, unless it has a large constituency paying in turn large dues to support it. There are too many journals in existence already, and state societies should not enter the field of competition, because it places them too distinctly in the commercial class, hence serves to lower their *esprit de corps*.

While the foregoing may not apply as forcefully in the west, where there are fewer journals, and among state societies that do not possess bound volumes showing nearly a hundred years of being, here in New York, with three splendid weeklies and a score or more of stalwart monthlies, it certainly has considerable application. Moreover, a journal in addition to the regular work of the society must print editorials, abstracts, foreign material, book reviews, and a mass of matter not germane to the real object of the society journal.

The salaries of an editor and a business manager also make a large item of expense which must be met monthly, all of which eats up the income with speed, to meet which larger dues are required than under the old plan. A bound volume is easy to find and consult; it is a book that will be cared for jealously, and be prized for the record it contains. For these and many other reasons that might be offered, we feel that it will be a mistake for the society to undertake the change from an annual volume to a monthly journal in the publication of its proceedings.

---

APOLLINARIS WATER has long been considered one of the most agreeable and popular table waters in use. Somebody has questioned its right to be classed as a natural mineral water, but this promptly was decided in the affirmative. The London *Lancet*, however, determined to investigate the subject for itself and recently sent a special commissioner to visit the Apollinaris spring in Germany. He reports the results of his investigations in the *Lancet* of January 30, 1904. It is based on numerous analyses made at the spring and of various samples of the water purchased in the open market. The article, which is long, interesting and conclusive, ends as follows:

It is difficult to suggest, in the face of the facts just recorded and of the experience which has decided upon the adoption of the methods of bottling Apollinaris which we have described, how those methods could be altered with any possible advantage to the public or how any modification of those methods would enable the public to receive the water in a condition more natural than it is. As a matter of fact Apollinaris water is bottled in such a way that the natural equilibrium of the water and its complement of gas at a depth 50 feet in the spring are preserved in the bottle for public use. Both water and gas are absolutely the natural products of the spring and the composition of the bottled water is, according to our analysis, always the same and without any appreciable variation in the mineral constituents. Some portion of a useless constituent, in the form of oxide of iron (the total amount in the water being quite minute) separates from the water prior to bottling but a useful constituent, in the shape of a small quantity of salt, is added to augment the amount of salt already naturally present in the spring in order to prevent the possible decomposition to which the sulphate of sodium of the water is occasionally liable. The taste of the water in bottle is identical with that of the water taken directly from the spring. Apollinaris water has a peculiar soft flavor which is due not to common salt at all, but in part to the alkaline carbonates which neutralise the acids in the mouth, and in part to the natural state of combination of the mineral ingredients. As Professor Oscar Liebreich has said, "even the best manufactured artificial mineral waters differ from the natural ones in taste and value." There is nothing disclosed in our analysis of the bottled water which is not found in the water at the spring. In view of these facts which we have taken some trouble to ascertain for ourselves, it seems to us that the recent decision of the Lord Chief Justice that Apollinaris water is entitled to the description of a natural mineral water is in accordance with both law and common sense.

We may add that our analyses and observations are in substantial agreement with those given at various times by the late Professor Virchow, Professor Bischof, Professor Liebreich, Professor Mohr, Professor Hofmann, Professor Kekulé, Professor William Odling, and the late Sir Edward Frankland.

Physicians will be pleased to observe the favorable comment of such high authority on this favorite water.

---

An epidemic of typhoid fever, at Watertown, N. Y., threatens to assume proportions of considerable magnitude. Several hundred cases have already been reported, though the percentage of deaths has been comparatively small. An expert from the state department of health has been sent to investigate the source of the epidemic which is supposed to lurk in the unfiltered water of the Black river. The lessons to be derived from the penalties paid by the neglect in other cities to maintain an uncontaminated water supply appear to be slow of inculcation.

---

A BILL to establish a laboratory for the study of the criminal, pauper, and defective classes is proposed to be introduced in the legislature at Albany. It is based on a similar bill already pending in congress. The importance of a study of these classes is recognised by scientific men all over the country. Such a bureau would have for its purpose the gathering of data of a sociological, pathological or abnormal nature and may well be undertaken by the state on economic grounds. As it is cheaper to prevent disease than to cure it, so it is cheaper to prevent crime than to punish it.

We are not disposed to enter into details relating to this subject at this time, our object being rather to invite attention to the propriety of establishing a systematic method of study relating to the classes mentioned, as an economic problem for the state to consider.

---

CLEAN money is an important desideratum as every physician will attest. Soiled currency is a medium for the transmission of disease; this, too, every physician knows. A remedy is offered in congress through a bill to create post check currency. In other words, it is proposed to create a system of bank notes, payable to the order of the individual named on the face of the bill. This can be sent through the mails, properly indorsed, similarly to personal checks or drafts. It is cancelled after one payment made through its medium, hence the danger of transmitting disease is reduced to a minimum. Fractional currency could thus be created which would meet a great demand in the payment of small sums to persons at a distance. This proposition being in the interest of the prevention of disease should receive the approval of every physician.

---

THE Bureau of Standards in the Department of Commerce and Labor at Washington, is created for the purpose of verifying standards relating to measuring instruments of various kinds. Among other things the testing of clinical thermometers will engage its attention. This important service will be appreciated by physicians and hereafter these thermometers will not be considered accurate unless the certificate of the bureau of standards is attached. This certificate will embrace the following data: (*a*) description of article or instrument; (*b*) Bureau of Standards test number, and certification number when allowed; (*c*) name of party for whom test is made; (*d*) temperatures at which comparisons are made; (*e*) other conditions of the test; (*f*) corrections at each test point; (*g*) date of certification; (*h*) seal of the Bureau and the signature of the director; (*i*) special remarks when necessary.

---

Dr. Ferd. C. Valentine, of New York, has had occasion to reprimand the A. S. Valentine Chemical Company through his attorney, for the use of the name *Valentine* in connection with a "Benzol-Capsule Valentine," which the company manufactures and markets under that commercial name. Dr. Valentine, in a published circular, says that the counselor of the so-called Valentine company had an interview with Mr. Quinby, Dr. Valentine's legal adviser, January 20, 1904, in the course of which the company's counsel promised to advise his clients to cease the use of the word Valentine in any manner whatever. In the circular referred to, Dr. Valentine asks to be notified if anyone shall discover any violation of this promise. His address is 31 West 61st street, New York.

